# An exclusive fine-needle biopsy approach to sampling solid lesions under EUS guidance: a prospective cohort study

**DOI:** 10.1093/gastro/goaa012

**Published:** 2020-04-15

**Authors:** Lindsey M Temnykh, Mahmoud A Rahal, Zahra Zia, Mohammad A Al-Haddad

**Affiliations:** Department of Internal Medicine, Indiana University School of Medicine, Indianapolis, IN, USA; Department of Internal Medicine, Indiana University School of Medicine, Indianapolis, IN, USA; Department of Internal Medicine, Indiana University School of Medicine, Indianapolis, IN, USA; Division of Gastroenterology and Hepatology, Department of Internal Medicine, Indiana University School of Medicine, Indianapolis, IN, USA

**Keywords:** endoscopic ultrasound, fine-needle biopsy, fine-needle aspiration, pancreatic tumors

## Abstract

**Background:**

Endoscopic ultrasound-guided fine-needle biopsy (EUS-FNB) is increasingly utilized to enhance the cytological yield of sampling solid lesions, but its superiority over existing fine-needle aspiration (FNA) platforms has not been clearly demonstrated. The aim of our study was to compare the diagnostic accuracy and procedural outcomes of FNB using a new Franseen-tip needle to that of a traditional FNA in sampling solid lesions under EUS guidance.

**Methods:**

Consecutive patients with solid lesions referred for EUS-FNB sampling were included. Procedure-related outcomes were collected prospectively including patient demographics, number of passes performed, diagnostic sample adequacy, adverse events, and recovery time. The Acquire needle was used to sample all lesions in the study group. Consecutive EUS-FNA procedures performed to sample solid lesions using the Expect needle were utilized as controls.

**Results:**

There were 180 patients undergoing EUS-FNB compared to 183 patients undergoing EUS-FNA procedures for solid-lesion sampling. The procedure time was significantly shorter in patients who underwent FNB compared to FNA (mean: 37.4 vs 44.9 minutes, *P* < 0.001). Significantly fewer passes were performed in the FNB cohort compared to the FNA group (mean: 2.9 vs 3.8, *P* < 0.001). The cytologic diagnostic yield was significantly higher in the FNB group compared to the FNA group (98.3% vs 90.2%, *P* = 0.003). No significant difference in the incidence of adverse events was observed between the FNB and FNA groups (1.1% vs 0.5%, *P* = 0.564).

**Conclusions:**

An FNB-exclusive approach to sampling solid lesions under EUS guidance is safe and feasible, and may result in fewer overall passes, shorter procedure time, and improved diagnostic adequacy. FNB may replace FNA as the primary sampling modality of choice in all solid lesions.

## Introduction

Endoscopic ultrasound-guided fine- needle aspiration (EUS-FNA) has been a standard modality used for the diagnosis and staging of gastrointestinal (GI) malignancies and other non-malignant lesions accessible from the GI tract. The diagnostic yield of EUS-FNA varies and remains dependent on a variety of factors including: the type, size, and location of the lesion; the sampling device; and the operator’s expertise [1, 2]. Endoscopic ultrasound-guided fine- needle biopsy (EUS-FNB) is increasingly being utilized to enhance cytological yield during the sampling of solid lesions [3]. A newer generation of FNB needles has helped to improve the tissue yield compared to the first generation of biopsy needles [4]. A meta-analysis showed similar diagnostic yield to standard FNA needles with fewer passes [5]. Our study compared the performance of a novel Franseen-tip biopsy needle to a standard FNA device in sampling solid lesions under EUS guidance.

## Patients and methods

### Study design and study population

This was a single-center study approved by the Institutional Review Board (Protocol #1703922591). Consecutive patients with solid lesions referred for EUS- guided sampling were included. All endoscopies were performed by a single endosonographer between September 2013 and March 2018 in a single high-volume outpatient endoscopy unit using anesthesia-administered propofol for the sedation of all patients. Patient demographics and lesion-related characteristics, including the size and location of target lesions, cytological and pathological findings, and clinical outcomes, were prospectively collected. Additional procedure-specific variables were also assessed, including procedure duration (from initiation to termination of sedation), sedative doses used, number of passes performed, diagnostic sample adequacy, overall recovery time, and adverse events up to 72 hours post procedure (including post-procedure hospitalizations and emergency- department visits).

### Techniques of EUS-FNB and EUS-FNA

**Figure 1.**
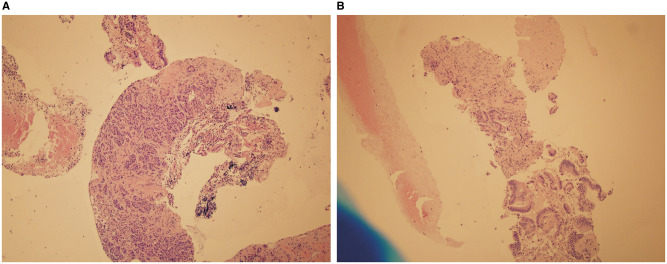
Fine-needle biopsy specimen obtained from pancreatic head masses. The lesions were sampled using a fine-needle biopsy technique. (A) The final diagnosis was pancreatic adenocarcinoma (Hematoxylin & eosin, ×10). (B) The final diagnosis was pancreatic adenocarcinoma with fibrosis (Hematoxylin & eosin, ×20).

**Figure 2.**
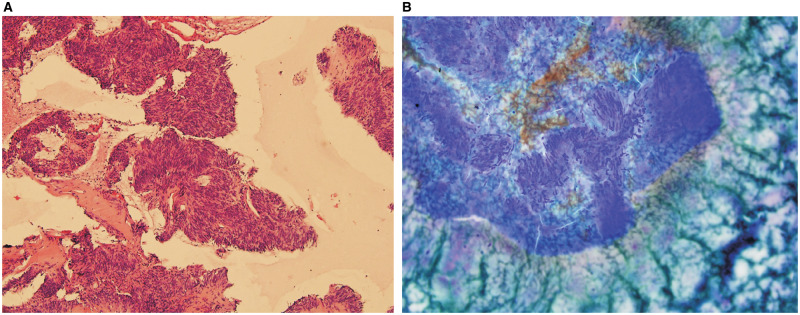
Fine-needle biopsy specimen obtained from gastric masses. The lesions were sampled using a fine-needle biopsy technique. (A) The final diagnosis was gastrointestinal stromal tumor (Hematoxylin & eosin, ×10). (B) The final diagnosis was gastrointestinal stromal tumor (Hematoxylin & eosin, ×20).

**Figure 3.**
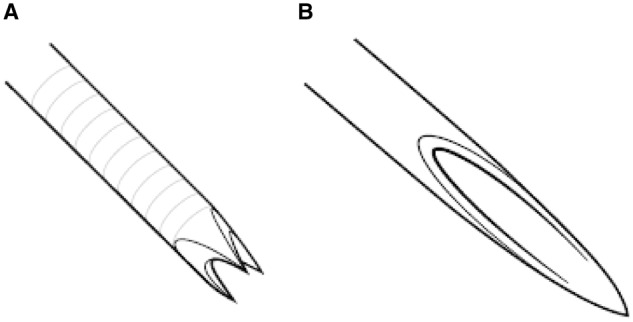
The Franseen needle design with the crown-shaped needle tip revealing the three symmetrical planes for histological core-tissue acquisition (A). The standard bevel needle used in fine-needle aspiration for the procurement of cytological aspirates (B).

The 22- or 25-gauge Acquire FNB needle (Boston Scientific, Natick, MA) was used to sample all lesions in the study group using a linear echoendoscope. This needle is a novel device with a Franseen- tip design with three symmetrical cutting edges. Lesions were punctured under EUS guidance and the stylet was removed and not replaced between passes. The fanning technique was routinely used and a low negative pressure (10 ml of suction) was used in all cases. Tissue was processed on-site with touch preps, with all excess visible cores placed in a cellular preservative for a cell block. Additional passes were allocated to the cell block based on the results of the rapid on-site evaluation (ROSE) and anticipated need for immunohistochemistry as specified by the endoscopist and the cytopathology team. This has been the standard of care at our center throughout the entire study period. Consecutive EUS-FNA procedures recently performed to sample solid lesions using the Expect needle (Boston Scientific, Natick, MA) were utilized as controls. Similar to FNB sampling, the fanning technique was used along with 10 ml of negative pressure in all cases. Tissue was processed on-site in the same fashion. FNB specimens were processed by cytology technicians, with all excess visible cores placed in a cellular preservative for a cell block (Figures 1 and 2). Additional passes were allocated to the cell block based on the results of the ROSE and anticipated need for immunohistochemistry. The diagnosis was typically rendered by the cytopathologist, who was not blinded to the needle used for the procedure. Procedure outcomes were abstracted from our clinical databases to allow head- to- head comparison with FNB outcomes. An illustration of the Franseen- tip needle and the standard bevel needle used in the study can be found in Figure 3.

### Study outcomes

Procedure-related outcomes were collected prospectively. The final diagnoses were based on unequivocal cytology provided by EUS and surgical pathology whenever performed. In cases with non-malignant outcomes, a subsequent clinical course was examined for ≤6 months to determine the outcomes and allow the assessment of the performance characteristics of the test and rule out false-negative FNB or FNA cases.

Our endoscopy- unit nurses routinely call all patients within 48–72 hours after the procedures to assess for short-term adverse events. Adverse events were recorded according to the published American Society of Gastrointestinal Endoscopy criteria [6]. Additional follow-up information after EUS was performed by reviewing medical charts to rule out adverse events developing beyond this window of time.

### Sample size and statistical analysis

Based on the literature, there was an expected 12% overall difference in the diagnostic yield between EUS-FNB (93%) and EUS-FNA (81%) when assessing all pancreatic and non-pancreatic lesions [7]. Using a confidence interval of 95%, we calculated a target sample size of 160 for each group. We included 180 patients in the FNB group and 183 patients in the FNA group. Descriptive analysis was used according to the type of variables used. A two-tailed distribution was used and a *P*-value  <0.05 was considered statistically significant. The categorical variables were measured as the count and percentage using the chi-square test. Continuous variables were measured as the mean and standard deviation using Student’s *t*- tests. Statistical analysis was carried out using IBM SPSS Statistics, version 23.0 (SPSS, Chicago, IL, USA).

## Results

### Patient characteristics

There were 180 patients in the FNB group and 183 patients in the FNA group included in the study. FNA was performed using a 22-gauge needle in 111 patients (69.5%) compared to 122 patients (71.8%) in the FNB group (*P* = 0.697). The mean age in the FNB group was 61.6 years vs 60.5 years in the FNA group (*P* = 0.106) and 45% of the FNB group were females compared to 47% in the FNA group (*P* = 0.777). Body mass index was slightly higher in FNB group compared to the FNA group (*P* = 0.030). Most of the patients belonged to American Society of Anesthesiologists (ASA) class 3 (Table 1).

**Table 1.** Baseline characteristics and final diagnoses of the 363 patients

Characteristic	FNB group (*n* = 180)	FNA group (*n* = 183)	*P*-value
Age, years, mean ± SD	60.5 ± 13	62.7 ± 12.4	0.106
Female, *n* (%)	81 (45.0)	86 (47.0)	0.777
BMI, kg/m^2^, mean ± SD	29.4 ± 7.4	27.7 ± 6.9	0.030
Weight, kg, mean ± SD	85.8 ± 23.5	79.9 ± 21	0.011
ASA class, *n* (%)			0.322
Class 2	62 (34.4)	58 (31.9)	
Class 3	115 (63.9)	119 (65.4)	
Final diagnosis			
Pancreatic lesions, *n* (%)	79 (43.9)	87 (47.5)	0.485
Adenocarcinoma	52 (65.8)	54 (62.1)	
Neuroendocrine tumor	11 (13.9)	11 (12.6)	
Chronic pancreatitis	7 (8.9)	2 (2.3)	
Non-pancreatic lesions, *n* (%)	101 (56.1)	96 (52.5)	0.708
Metastatic cancer	9 (8.9)	2 (2.1)	
Adenocarcinoma	25 (24.8)	30 (31.3)	
Gastrointestinal stromal tumor	7 (6.9)	8 (8.3)	
Neuroendocrine tumor	3 (3.0)	3 (3.1)	
Leiomyoma	4 (4.0)	2 (2.1)	
Lymphoma	4 (4.0)	3 (3.1)	
Reactive lymphadenopathy	6 (5.9)	9 (9.4)	

FNB, fine-needle biopsy; FNA, fine-needle aspiration; SD, standard deviation; BMI, body mass index; ASA, American Society of Anesthesiologists.

### Pathologic findings

There were 87 solid pancreatic lesions in the FNA group vs 79 in the FNB group (*P* = 0.485), with pancreatic adenocarcinoma being the most common diagnosis (65.8% in the FNB group vs 62.1% in the FNA group). Neuroendocrine pathology was the second most common type of pancreatic tumor (13.9% and 12.6% in the FNB and FNA groups, respectively). There were 101 non-pancreatic lesions in the FNB group vs 96 in the FNA group (*P* = 0.708), mainly including metastatic lesions, non-pancreatic adenocarcinomas, gastrointestinal stromal tumors, neuroendocrine tumors, lymphoma, leiomyomas, and reactive lymphadenopathy (Table 1). In both groups, almost half of the pancreatic biopsies (80/166) involved the head or uncinated process. The non-pancreatic lesions were present in the stomach, esophagus, small and large intestines, liver, and biliary tree. Among non-pancreatic lesions, 68 (43.6%) were benign while 88 (56.4%) lesions were malignant.

### Procedure characteristics

The mean procedure time was significantly longer in the FNA group compared to the FNB group (44.9 vs 37.4 minutes, *P* < 0.001). There was no significant difference in antibiotics use, total dose of propofol used, and recovery time between the FNA and FNB groups (Table 2).

**Table 2.** Characteristics and outcomes of the FNA and FNB procedures under EUS guidance

Characteristic	All patients (*n* = 363)	FNB group (*n* = 180)	FNA group (*n* = 183)	*P*-value
Antibiotics use, *n* (%)	65 (17.9)	30 (16.7)	35 (19.1)	0.574
Procedure time, minutes, mean ± SD	41.2 ± 14.1	37.4 ± 13.2	44.9 ± 14.1	<0.001
Propofol dose, mg, mean ± SD	459 ± 196	453 ± 189	465 ± 204	0.585
Recovery time, minutes, mean ± SD	67.5 ± 33.7	65.4 ± 38.5	69.4 ± 28.3	0.264
Number of passes, mean ± SD	3.4 ± 1.8	2.9 ± 1.1	3.8 ± 2.1	<0.001
More than two needle passes, *n* (%)	225 (62.7)	103 (57.2)	122 (66.7)	0.111
Adequate diagnostic specimen, *n* (%)	340 (94.2)	177 (98.3)	165 (90.2)	0.003
Adverse event, *n* (%)	3 (0.8)	2 (1.1)	1 (0.5)	0.564

FNB, fine-needle biopsy; FNA, fine-needle aspiration; EUS, endoscopic ultrasound; SD, standard deviation.

### Diagnostic yield and number of passes

The cytologic diagnostic yield was significantly higher in the FNB group at 98.3% compared to 90.2% in the FNA group (*P* = 0.003; Table 2). In subgroup analysis for pancreatic lesions, the diagnostic yield was significantly higher in the FNB group compared to the FNA (98.7% vs 92.0%, *P* = 0.042). After stratifying patients based on the location of the lesion sampled, the diagnostic yield was still higher among the pancreatic head or pancreatic body/tail in the FNB group vs the FNA group, but the differences did not reach statistical significance. Among non-pancreatic lesions, there was no significant difference in the diagnostic yield between the FNA and FNB groups based on location (Table 3).

**Table 3.** Subgroup analysis of diagnostic yield and number of passes based on location

Location	Adequate diagnostic specimen, *n* (%)			Number of passes, mean ± SD		
	FNB	FNA	*P*-value	FNB	FNA	*P*-value
Solid pancreatic masses	*n* = 79	*n* = 87		*n* = 79	*n* = 87	
Pancreatic lesions	78 (98.7)	80 (92.0)	0.042	3.1 ± 1.3	3.9 ± 2.1	0.009
Pancreatic head	36 (97.3)	39 (90.7)	0.224	3.4 ± 1.4	3.9 ± 2.4	0.343
Pancreatic body/tail	42 (100)	41 (93.2)	0.085	2.9 ± 1.0	3.9 ± 1.8	0.003
Non-pancreatic masses	*n* = 101	*n* = 96		*n* = 101	*n* = 96	
GIST and leiomyomas	15 (88.2)	13 (86.7)	0.893	3.0 ± 1.3	5.0 ± 1.7	0.002
Mediastinal/esophageal	26 (96.3)	27 (90)	0.353	3.3 ± 1.3	3.6 ± 2.1	0.618
Gastric mass	16 (100)	14 (82.4)	0.078	3.0 ± 1.1	3.8 ± 1.9	0.163
Small/large intestine	10 (90.9)	18 (94.7)	0.685	3.4 ± 1.1	3.6 ± 1.7	0.651
Liver	32 (100)	9 (100)	–	2.1 ± 0.5	2.3 ± 1.0	0.403
Bile duct	5 (100)	8 (80)	0.283	2.4 ± 0.5	5.3 ± 2.8	0.039

SD, standard deviation; FNB, fine-needle biopsy; FNA, fine-needle aspiration; GIST, gastrointestinal stromal tumor.

The mean number of passes was significantly lower among patients in the FNB group compared to the FNA group (2.9 vs 3.8, *P* < 0.001). When considering pancreatic lesions only, the average number of passes remained significantly lower in the FNB group (*P* = 0.009; Table 3).

### Adverse events

No significant difference in the incidence of adverse events was observed between the FNB group and the FNA group (1.1% vs 0.5%, *P* = 0.564). One patient in the FNA group experienced severe post-procedural pain requiring admission without identified perforation or pancreatitis while two patients in the FNB group were hospitalized for post-procedural pancreatitis in one and pain management in another, with <3 days of hospitalizations each. No interventions or surgery was required in any patient. All three adverse events were classified as ‘mild ’ based on the American Society for Gastrointestinal Endoscopy lexicon for adverse events.

## Discussion

EUS-FNA has been the standard for the evaluation and sampling of solid pancreatic and non-pancreatic lesions that can be sampled across the gut wall. EUS-FNA has been limited in the adequacy of diagnostic specimens, even in the setting of ROSE [8, 9]. The sample adequacy of EUS-FNA has ranged from 52% in non-pancreatic lesions to 89% in pancreatic lesions [3, 10, 11]. Sample adequacy is known to be impacted by a variety of factors, including the availability of ROSE , experience of the endosonographer, location and characteristics of the lesion, and the tissue- procurement method [12]. Sample adequacy is critical to establish a diagnosis of malignancy, advance treatment plans, and avoid delays related to repeat EUS and sampling [5].

EUS-FNB has been increasingly utilized as an alternative to EUS-FNA to improve diagnostic adequacy, particularly in challenging lesions where cytology from previous FNA was inconclusive or further tissue assays are necessary. In a study by Aadam *et al*. [11], EUS-FNB was found to have significantly higher diagnostic yield compared with EUS-FNA, although, in subgroup analysis, there was no significant difference in the diagnostic yield for pancreatic lesions. In addition, a recent meta-analysis demonstrated no significant difference in the diagnostic adequacy (75% vs 89%), diagnostic accuracy (85% vs 86%), or rate of histological core- specimen acquisition (78% vs 77%) between the ProCore (Cook Medical, Winston-Salem, NC) and standard FNA needles [5]. To date, prior studies have consistently demonstrated a reduced number of passes associated with EUS-FNB compared to EUS-FNA, although studies have been inconsistent on whether EUS-FNB is superior to FNA when it comes to sample adequacy and procedure time [7, 13, 14]. Additionally, the diagnostic yield was found to be significantly greater for FNB compared with FNA when evaluating sub-epithelial lesions in one study [14].

Our study aimed at assessing a new core- biopsy device with three cutting surfaces and a Franseen-shaped tip [15]. We demonstrated in this report that this device provides histologically superior samples with fewer passes and decreased procedure time compared to conventional FNA needles of the same size. To our knowledge, this is the first report to demonstrate the advantage of reduced procedure time associated with more efficient routine utilization of EUS-FNB sampling in solid lesions, likely related to the reduced number of passes. Furthermore, in subgroup analysis, EUS-FNB had a greater diagnostic adequacy in the pancreas overall when compared to EUS-FNA. Although previous studies showed that FNB has better diagnostic accuracy achieved by fewer needle passes compared to FNA in evaluating gastrointestinal stromal tumors (GIST) and leiomyomas [14, 16], we did not observe a significant difference in the diagnostic accuracy in this group of patients. In addition, there was no significant difference in the incidence of adverse outcomes between the FNA and FNB groups.

Our study has some limitations. This is a retrospective study using prospectively collected data in a high- volume referral center. An ideal design to compare the sampling capabilities of two needle platforms would entail a randomization scheme. Although our study was not designed to be a randomized–controlled trial, we have powered it to allow the adequate assessment of several end points related to the sampling process and pathology yield. In addition, we attempted to standardize as many variables known to impact the outcomes of EUS-guided sampling as possible to reduce bias. For example, we used standard FNA and FNB sampling techniques performed by the same endosonographer, using standard cytological processing techniques as previously reported when processing FNB samples [15]. In addition, we have relied on the same sedation methods (anesthesia- administered propofol) by the same group of anesthesia providers for all cases included to avoid variabilities in style that can impact the overall procedure time and propofol dose used. This way, we have retained all the elements of real-time endosonography practice that apply to academic and community practices alike.

The question of whether the various EUS-FNB devices deliver the same quality of tissue specimens remains to be answered. The bulk of the published research on FNB to date has assessed the performance of one line of reversed- bevel needles (Procore, Cook Medical, Winston-Salem, NC). A meta-analysis failed to demonstrate a clear advantage of that needle over the traditional FNA [5]. Since the introduction of Procore, two additional needles with unique tip designs became available. Our study is one of a few demonstrating the increased diagnostic accuracy of a relatively new Franseen-tip FNB platform [15, 17, 18].

In summary, an FNB-exclusive approach to sampling solid lesions under EUS is feasible and results in fewer overall passes, increased diagnostic adequacy, and decreased procedure time, and may obviate the need for an on-site pathology review given the high diagnostic yield. FNB may replace FNA as the primary sampling modality of choice in all solid lesions. Further studies are needed to further characterize the performance of this device in lesions of various origins and locations. In addition, further studies comparing the Acquire FNB needle to other available FNB devices would be helpful to determine the most effective FNB device.

## Authors’ contributions

L.M.T.: literature search, data acquisition, manuscript preparation, manuscript editing. M.A.R.: literature search, data acquisition, data analysis, manuscript preparation, manuscript editing. Z.Z.: data acquisition. M.A.A.: concept, design, literature search, data acquisition, manuscript editing. All authors read and approved the final manuscript.

## Funding

No funding was received for this project. 
